# Dietary Mycotoxins: An Overview on Toxicokinetics, Toxicodynamics, Toxicity, Epidemiology, Detection, and Their Mitigation with Special Emphasis on Aflatoxicosis in Humans and Animals

**DOI:** 10.3390/toxins16110483

**Published:** 2024-11-08

**Authors:** James Kibugu, Leonard Munga, David Mburu, Fredrick Maloba, Joanna E. Auma, Delia Grace, Johanna F. Lindahl

**Affiliations:** 1 Biotechnology Research Institute, Kenya Agricultural and Livestock Research Organization, P.O. Box 362, Kikuyu 00902, Kenya; joanna.auma@kalro.org; 2 Department of Biochemistry, Microbiology and Biotechnology, School of Pure and Applied Sciences, Kenyatta University, P.O. Box 43844, Nairobi 00100, Kenya; nmburu01@gmail.com; 3 Department of Animal Science, School of Agriculture and Environmental Sciences, Kenyatta University, P.O. Box 43844, Nairobi 00100, Kenya; munga.leonard@ku.ac.ke; 4 Department of Zoological Sciences, School of Pure and Applied Sciences, Kenyatta University, P.O. Box 43844, Nairobi 00100, Kenya; malobafred@gmail.com; 5 Department of Biosciences, International Livestock Research Institute, P.O. Box 30709, Nairobi 00100, Kenya; d.randolph@cgiar.org; 6 Natural Resources Institute, University of Greenwich, UK, Central Avenue, Chatham ME4 4TB, UK; 7 Department of Animal Health and Antibiotic Strategies, Swedish Veterinary Agency, 75189 Uppsala, Sweden; johanna.lindahl@sva.se; 8 Department of Medical Biochemistry and Microbiology, Uppsala University, 75123 Uppsala, Sweden

**Keywords:** mycotoxins, epidemiology, aflatoxin, toxicology, toxicity, detection, control strategies

## Abstract

Mycotoxins are secondary metabolites of filamentous fungi and ubiquitous dietary contaminants. Aflatoxins, a group of mycotoxins with high prevalence and toxicity, have raised a high level of public health concern, the most prevalent and toxic being aflatoxin B1 (AFB1). Many aspects appertaining to AFB1 poisoning are not well understood. Yet this information is necessary to devise appropriate surveillance and mitigation strategies against human and animal aflatoxicosis. This review provides an in-depth update of work carried out on mycotoxin poisoning, particularly aflatoxicosis in humans and animals, to identify gaps in knowledge. Hypotheses explaining the functional significance of mycotoxins in fungal biology and their dietary epidemiological data are presented and briefly discussed. The toxicology of aflatoxins and the challenges of their mitigation are discussed in depth. It was concluded that the identification of potential mycotoxin-hazard-prone food items and quantification of the associated risk of cancer ailments in humans is a prime priority. There is a dearth of reliable sampling methodologies for estimating AFB1 in animal feed. Data update on AFB1 in animal feed and its implication in animal production, mitigation strategies, and elucidation of risk factors to this hazard is required. To reduce the burden of aflatoxins, surveillance employing predictive technology, and biocontrol strategies seem promising approaches.

## 1. Introduction

In developing countries, the consumption of unsafe food is a major cause of preventable disease and mortality in both humans and animals [1]. Food products are susceptible to contamination at origin and as they move along the value chain, exposing consumers to health hazards; this is compounded by rapidly changing production practices, a lack of human and institutional capacity, poor oversight by governments, and limited public health awareness [2,3]. Mycotoxins can enter the food/feed chain during any stage of the value chain. Mycotoxins have been ubiquitous in the tropical world for a very long time, the highest burden being in sub-Saharan Africa (SSA), South East Asia (SEA), and China [4,5,6]. Mycotoxicosis is among the contributory factors to the seemingly increased incidence of cancers, especially those associated with aflatoxins, such as hepatocellular carcinoma [2,5,7,8,9,10]. It has recently been suggested that dietary tremorgenic mycotoxins may be a significant cause of neurological diseases in both humans and animals [11].

Dietary mycotoxins are natural contaminants that are difficult to avoid [5,12]. The diagnosis of the array of toxic effects they induce in humans and animals after consumption is also difficult. Many are silent diseases that escape the notice of medical and veterinary personnel, the only readily available diagnostic tools being those for the detection of dietary mycotoxins by chemical analytical methods. However, accurate estimation of dietary aflatoxin contamination has been problematic due to its heterogeneity [13,14,15,16,17]. Although there have been achievements in the improvement of the analytical characteristics of detection methods [18,19,20], uncertainty associated with aflatoxin estimation is largely associated with sample selection and preparation procedures [14,17,21,22]. Indeed, obtaining a representative sample for aflatoxin analysis is problematic [23,24,25].

Dietary mycotoxicosis, especially aflatoxicosis, compromises health and performance, reduces vaccine efficacy in animals, and exposes consumers to violative mycotoxin residues in edible animal products [26,27,28,29,30]. Indeed, in poultry, experimental aflatoxicosis impairs production parameters, induces pathology, reduces egg production, and introduces aflatoxin residues in tissues and eggs [26,28,31,32,33,34]. Likewise, aflatoxins and other mycotoxins in food items also cause human morbidity [5,8,12]. Mycotoxins have an array of effects on human health [35], the most important being their contribution to primary hepatocellular carcinoma and immunosuppression by aflatoxins [2,9,10]. One of the most promising control strategies to curb dietary animal mycotoxicosis is the amelioration of mycotoxin effects in animals through the use of dietary anti-mycotoxin agents [36,37,38]. This is an in-feed approach where an anti-mycotoxin material is incorporated into the feed to sequestrate by adsorption, binding, or neutralizing the mycotoxin residues. A variety of these dietary additives are commercially available on the market. However, the use of these feed additives is not officially regulated, and therefore, their in vivo efficacy in terms of anti-mycotoxin activity is seldom validated. This means that, despite their official approval for use, their benefits to animal production relating to the counteraction of aflatoxicosis outcomes are largely unknown, an ambiguity that confuses the stakeholders of animal value chains. The purpose of this review is to provide an in-depth update of work carried out on the epidemiology, toxicokinetics, toxicodynamics, detection, and management of dietary mycotoxin poisoning, particularly human and animal aflatoxicosis, and also identify gaps in knowledge for future research.

## 2. Mycotoxins

Filamentous fungi produce secondary metabolites known as mycotoxins. They are ubiquitous natural contaminants of human food, animal feed, and agricultural products, the most commonly encountered being aflatoxins, ochratoxins, zearalenone (ZEA), fumonisins, trichothecenes, and patulin, which are produced by the fungal genera *Aspergillus*, *Penicillium,* and *Fusarium* [12,39,40,41,42,43,44,45]. Currently, the functional significance of these molecules is speculative and elusive [46,47]. Some proposed functions of mycotoxins are the attainment of a competitive advantage of the producer fungus over other microbiota in the ecological niche and anti-fungivore insect activity in the trophic niche [47,48,49]. Another is the potentiation of fungal invasion in plant hosts through mimicking plant effector molecules modulating plant growth or inducing programmed cell death and tissue necrosis to access plant nutrients for fungal utilization [50]. Recent data suggest that in aflatoxigenic fungi, aflatoxins are antioxidants, in other words, natural scavengers of reactive oxygen species (ROS), an adaptation that enhances fungal survival under oxidative stress (OS) conditions [51,52].

### Epidemiological Studies of Dietary Mycotoxins: A Brief Overview

Due to a favorable climate for fungal growth and inadequate regulation, outbreaks of acute mycotoxicosis involving deaths are common in SSA, SEA, South Asia, and China along tropical and sub-tropical zones [35,53,54]. There have been frequent reports of acute aflatoxicosis in Uganda, India, Kenya, Tanzania, Taiwan, Malaysia, and the USA, among other countries [5,6,55]. In Kenya, there have been 10 documented episodes of acute aflatoxicosis in humans and 5 in animals, with the severest being in 2004, which resulted in 317 cases and 125 mortalities of humans [6,56,57,58,59]. In SSA, exposure to mycotoxins is largely underreported, with few scanty data, mostly chronic, and characterized by co-occurrence of multiple mycotoxins with possible synergistic interaction [19,60,61]. These contaminants are widespread in human food and animal feed matrices in this region [9]. In Uganda, Wokorach et al. [60] reported a high prevalence of aflatoxins, fumonisins, ochratoxins, and deoxynivalenol (DON) co-occurring in sorghum, maize, groundnut, and millet, and their daily intakes exceeding regulatory thresholds. Southern Africa had aflatoxins in maize and peanut products, fumonisin in maize products, and patulin in apple juice [62]. High AFB1 levels with large portions of samples surpassing regulatory thresholds were detected in maize and groundnuts from Tanzania [10,63] and maize from Rwanda [64]. In Kenya, a high prevalence of aflatoxin, ochratoxin A (OTA) in rice, and aflatoxin and fumonisin in maize with large non-compliance to regulatory standards were observed [9,65,66]. Indeed, surveys conducted in Kenya between 1960 and 2018 show high rates of non-compliance to dietary aflatoxin thresholds for maize, peanuts, sorghum, and milk products and astronomically high for animal feed [65,67,68]. Mycotoxin contamination has also been reported in both fruit juices and dried fruits [20,54,62]. Of particular health concern are aflatoxin hazards in dried fruits, which are associated with toxigenic fungal contamination, especially during the drying process [5,10,20,54,69]. Aflatoxin residues in milk and cereals in Kenya, notably important components of weaning and children’s foods [9,70,71,72,73] and maternal blood at delivery in Kenya and Nigeria [6], as well as fumonisin in breast milk in Ethiopia [74], indicate pediatric and infantile exposure.

In feed analyzed globally, aflatoxins, fumonisins, DON, ZEA, and OTA, largely in co-occurrence, have been detected [69]. In poultry feed, levels above regulatory thresholds of multiple mycotoxins, most prominently fumonisin B1 (FB1) and AFB1 in Nigeria [75], ZEA, fumonisin, DON, OTA, and aflatoxins in South Africa [76], and aflatoxin in Uganda and Cameroon were observed [20,77]. Peanuts and maize are aflatoxin-high-risk feed ingredients for the preparation of poultry feed [75,77]. Co-occurrence of AFB1, FB1, ZEA, and DON in fish feed from Nigeria was reported [78]. In Kenya, Rodrigues et al. [79] reported B-trichothecenes, fumonisins, ZEA, aflatoxins, and OTA residues in animal feed, which, particularly for aflatoxin, were above regulatory limits. AFB1, DON, ergot alkaloids, fumonisins, HT-2 toxin, OTA, T-2 toxin, and ZEA are mycotoxins that contaminate feed raw materials, and finished dairy feed and poultry feed [19,70,72]. Frequently, there is concurrent contamination with a high non-compliance rate for aflatoxin content [19]. In Kenya, aflatoxin content was detected in commercial broiler feed, with large portions of samples surpassing FAO/WHO and FDA regulatory limits [80]. There is also a gap in knowledge/or scanty information on the prevalence of dietary non-aflatoxin mycotoxins, albeit their food safety significance. Again, there is scant information on aflatoxin in food and feed items other than maize, particularly for broiler feed, whose prevalence of aflatoxin residues data are inadequate and outdated.

## 3. Aflatoxins

Because of their wide prevalence and toxicity, aflatoxins have raised public health concerns [4,5]. They were first discovered in poultry by British scientists in 1961 when more than 100,000 turkeys and other farm animals died from a condition then termed Turkey ‘X’ disease in the United Kingdom, whose cause was later found to be aflatoxins in animal feed originating in Brazil [81,82]. Aflatoxins are produced by *Aspergillus* fungi [83]. Some aflatoxin-producing species are *A. flavus*, *A. parasiticus*, *A. nomius*, *A. minisclerotigenes*, *A. arachidicola,* and *A. australis,* whose aflatoxigenic strains are found in agricultural commodities, food, and feed [5,46,51]. Albeit numerous aflatoxigenic fungi among the *Aspergillus* species, only *A. flavus* and *A. parasiticus* are of public health significance [84].

### 3.1. Types

Several aflatoxin molecules naturally occur as fungal or animal metabolites, namely aflatoxin B1 (AFB1), AFB2, AFB2a, AFP1, AFQ1, AFQ2a, aflatoxicol (AFL), AFL H1, AFL M1, AFG1, AFG2, AFG2a, AFM1, AFM2, AFGM1, AFGM2, AFGM2a, AFB3 (parasiticol), aspertoxin, and AFB1 8,9 epoxide [83,85]. The four primary types designated as aflatoxin B1, B2, G1, and G2 [86], together with AFB3 and aspertoxin, are natural dietary contaminants of plant-based food matrices [22], while AFM1, P1, Q1, aflatoxicol, and AFGM1 are carryover contaminants in either animal-based food items, human and animal excretory products, or animal and breast milk [83,87,88,89,90]. *A*. *flavus* produces B-type aflatoxins, while s*A*. *parasiticus* secretes both B- and G-aflatoxin types [87], the most prevalent and toxic being AFB1 [85,91]. B-aflatoxins or G-aflatoxins are primary aflatoxins. The letters “B” and “G” derived, respectively, from blue (425 nm) and green-blue (540 nm) fluorescence emission under ultraviolet (UV) light by the toxins isolated by thin-layer chromatography, and “1” or “2” denote a major or minor molecule, respectively [92,93,94].

### 3.2. Nomenclature and Structure

**Figure 1:**
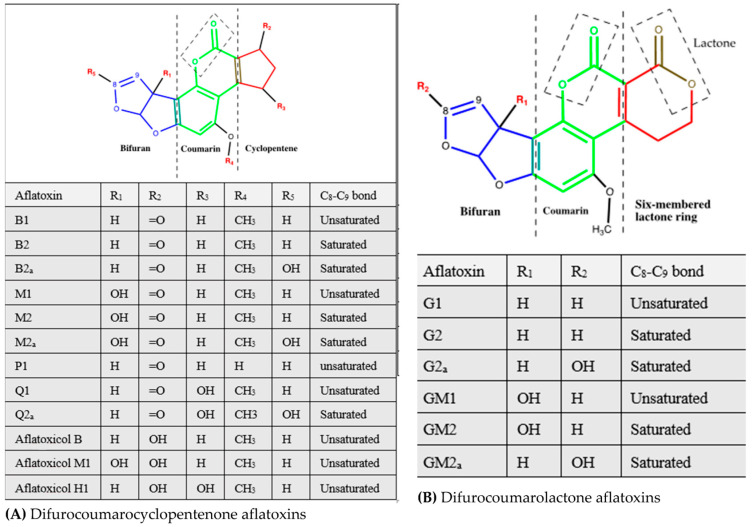
Basic structures of primary aflatoxins. (**A**) Difurocoumarocyclopentenones: B-aflatoxins; (**B**) difurocoumarolactones: G-aflatoxins. The bifuran moiety (highlighted in blue) associated with AFB1, G1, and other aflatoxins have an unsaturated C8=C9 double bond, which is prone to enzymatic insult (bio-activation), conferring the molecule’s high toxicity and carcinogenicity. The backbone of the molecule is the coumarin nucleus (shown in green). Highlighted in red are cyclo-pentene ring (for difurocoumarocyclopentenones) and lactone ring (for difurocoumarolactones). (Source: Benkerroum [5]).

By chemical structure, B-aflatoxins, AFB2a, AFP1, AFQ1, AFQ2a, aflatoxicol, aflatoxicol H1, and aflatoxicol M1 are classified as “difurocoumarocyclopentenones,” characterized by a fusion of the cyclopentenone ring to the lactone ring of the coumarin structure, and G-aflatoxins, AFG1, AFG2, AFG2a, AFGM1, AFGM2, AFGM2a, are designated “difurocoumarolactones” [83,93,95]. As shown in Figure 1, the skeleton structure of primary aflatoxins is a 3-component molecule comprising of a coumarin nucleus, a difuran moiety, and either a cyclo-pentene ring for B-aflatoxins or a six-sided lactone ring for G-aflatoxins, giving a categorizing criterion for difurocoumarocyclopentenones and difurocoumarolactones groups of aflatoxins, respectively [5,83]. AFB1, a difurocoumarocyclopentenone, has an unsaturated furan moiety with a highly reactive C8=C9 double bond, potentiating the molecule to activation by CYP450 enzymes [5]. The furan ring and coumarin group, in particular the lactone moiety, are vital for the toxicity of aflatoxin molecules and targets of degradation and detoxification.

### 3.3. Physical and Chemical Properties

Aflatoxins form clear to pale-yellow crystals and are fluorescent intensely under UV light [35]. Values of spectral characteristics could vary depending on factors such as solvent environment. For AFB1, the maximum fluorescence spectrum is 365 nm for excitation and 425 nm for emission in trichloroethylene/chloroform [86,96,97]. AFB1 is insoluble in non-polar solvents, slightly soluble in water (10–30 μg/mL), freely soluble in moderately polar organic solvents (chloroform and methanol), and particularly dimethyl sulfoxide [35,98]. The chemical properties of AFB1 are a molecular weight of 312.06 g/mol, a chemical formula of C_17_H_12_O_6,_ and a melting point of 268.5 °C [5,35,96]. It has strong thermal stability even above 100 °C but is unstable to UV light or pH conditions below 3 and above 10 [98].

### 3.4. Toxicokinetics

**Figure 2:**
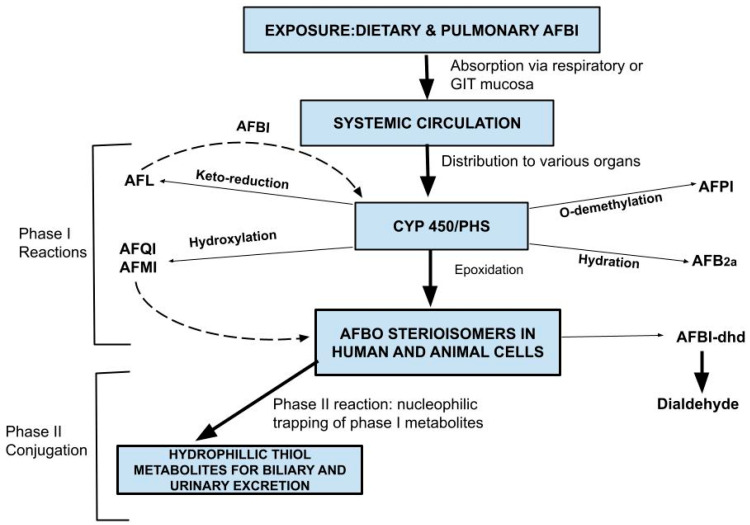
Schematic illustration of toxicokinetic events of AFB1 in animals and humans after exposure via oral and respiratory routes. The toxin is absorbed through mucosal cells, distributed to various body compartments, and undergoes phase I and II reactions. The hallmark of this pathway is the bio-activation of AFB1 into highly reactive electrophile AFBO.

Toxicokinetics involves physicochemical processes of absorption (A), distribution (D), metabolism/biotransformation (M), and excretion/elimination (E) of a toxicant by the body, a paradigm abbreviated as ADME [99,100] or LADME when liberation (L) for accessibility of the toxin from a matrix such as food is involved [101,102]. For AFB1, ADME processes follow first-order kinetics; that is, the rate of each toxicokinetic segment is proportional to the toxicant level [35,103]. Figure 2 illustrates the toxicokinetic events of AFB1 in humans and animals.

#### 3.4.1. Absorption

Important determinants of absorption are size, lipophilicity, hydrophobicity of guest molecules, and the age of the host organism, among other factors [103,104]. Aflatoxins have low molecular weight and high liposolubility, predisposing them to rapid absorption through the mucosa of the respiratory tract by inhalation or the gastrointestinal tract (GIT) through the oral route via a non-elucidated passive mechanism into mesenteric venous blood [35,93,95,103,105]. Indeed, aflatoxin can also be absorbed via vaginal mucosa, suggesting passive diffusion as the probable mechanism of absorption [105]. Other experimental routes of aflatoxin administration are percutaneous, intratracheal, intraperitoneal, and intraduodenal instillation applications, which deliver the toxin to the blood system [35,103,104]. Oral/dietary intake is the most important route of exposure to mycotoxins, including aflatoxins [101], and, therefore, the focus of the present study.

A review of toxicokinetic studies employing radiometric methods identified small intestines as an important site of toxin absorption where rapid first-order passive diffusion of AFB1 into mesenteric venous blood occurs; the duodenum is the most competent region of toxin uptake [103]. Age-related variation due to differences in intestinal epithelium lipid composition, with young animals having higher uptake than older ones, was also noted, confirming lipophilicity as a determinant of aflatoxin absorption. Further, there was evidence of a hepatoprotective strategy involving enteric first-pass effect biotransformation in gastrointestinal mucosa during AFB1 absorption [103]. Cows orally exposed to AFB1 at 4 μg/kg b.w., followed by another of 40 μg/kg b.w., showed a C_max_ of 3.8 ng/mL and a T_max_ of 35 min [89]. AUC, T_max_, and C_max_ values showed species variation due to differences in AFB1 intake, gastrointestinal absorption, animal health, and especially the activity of CYP450 enzymes. In humans exposed to dietary AFB1, T_max_ was about 1 h [35]. These data indicate rapid absorption of AFB1 via the GIT into the systemic circulation.

#### 3.4.2. Distribution

Once in the systemic circulation, AFB1 is distributed to various tissues, including the liver, its main target organ, and the site of xenobiotic metabolism [93,101], with similar patterns after intratracheal instillation and oral administration in rats [35]. The distribution process is influenced by the volume of distribution (v_d_, an index of affinity between a toxicant and tissues), plasma protein binding, tissue accumulation (the partition between blood and specific tissues), and permeability across specialized physiological barriers [103,104]. A review by Hsieh and Wong [103] observed that a high value of v_d_ indicates extensive tissue toxicant sequestration and increased species susceptibility to AFB1 toxicity. Toxin residue levels rapidly decrease in the kidney and liver, with most of the hepatic retained AFB1 being irreversibly bound to tissue macromolecules, especially serum albumin at the site of intestinal absorption, a vital detoxification mechanism [103].

#### 3.4.3. Metabolism

AFB1 undergoes activation (phase I) and conjugation (phase II) reactions [92,103], mainly via the mercapturic acid pathway [106]. Phase I metabolism involves oxidation (peroxidation, hydroxylation, o-demethylation), ketoreduction, and hydrolysis. AFB1 is mainly bio-transformed by hepatic and extra-hepatic membrane-bound cytochrome P450 (CYP450) microsomal monooxygenase enzymes to hydroxylated (AFM1, AFQ1), hydrated (8-hydroxy derivative, AFB2a or hemiacetal), o-demethylated (AFP1) metabolites, and epoxidated to AFB1-endo and -exo (8, 9) epoxides, or reduced via AFB1 reductase to aflatoxicol, AFL [87,89,92,107,108]. AFL is considered a storage form as it can be oxidized back to AFB1 by AFL dehydrogenase [94,97]. AFP1 can further be hydroxylated to 4,9a-dihydroxyaflatoxin B1 (AFM1-P1) [97]. Both exo- and endo-epoxides can be detoxified by rapid spontaneous or epoxide hydrolase (EPHX)-catalyzed hydrolysis to AFB1-8,9 dihydrodiol (AFB1-dhd), which undergoes furofuran ring opening to a dialdehyde (AFB1 α-hydroxydialdehyde) to form protein adducts by undergoing Schiff-base formation with lysine in serum albumin [97,109,110]. The dialdehyde can further be detoxified to a dialcohol through reduction catalyzed by aflatoxin aldehyde reductase (AFAR) [41,93,97,110]. Hepatic and extra-hepatic activation of AFB1 is also mediated by prostaglandin H synthase (PHS) and lipoxygenases [111,112] or CYP3A enzymes in enterocytes and lipoxygenase and PHS in kidneys and lungs [41,113,114]. Phase II reactions include glutathione *S*-conjugation, glucuronidation, sulfonation, acetylation, methylation, and amino acid conjugation of the parent compound or its Phase I metabolites, respectively, mediated by glutathione-*S*-transferases (GSTs), uridine 5′ diphosphate (UDP)-glucuronosyltransferases (UGTs), sulfotransferases (SULTs), acetyltransferases (NATs), methyltransferases, and aminoacyl-tRNA synthetase enzymes [41,97,115]. This is the principal detoxification pathway of AFBO via conjugation to the antioxidant glutathione (GSH) through a nucleophilic trapping process where electrophilic AFBO is conjugated with GSH in a reaction mediated by GSTs [85,97]. This conjugate undergoes hydrolysis and N-acetylation, forming a hydrophilic species, mercapturic acid (aflatoxin-mercapturate) [106,115]. AFM1, AFQ1, AFP1, and aflatoxin M1-P1 and aflatoxicol are conjugated with uridine diphophate (UDP)-glucuronic acid (glucuronidation) and sulfates (sulfonation) in reactions catalyzed by UGTs and SULTs, respectively [35,87,97,103,116].

#### 3.4.4. Excretion

A toxicant is excreted either as a parent molecule or its metabolites [99]. The toxicokinetic modeling parameters for excretion are elimination half-life (t_1/2_), elimination rate constant (k_el_), and clearance (CL), particularly renal clearance [99,117,118]. CL is the volume of blood from which a toxicant is irreversibly eliminated or cleared per unit of time, while t_1/2_ is derived from k_el_ [99,119]. Oral administration of AFB1 in rats at 0.72, 18.1, and 600 µg/kg body weight yielded k_el_ of 0.01 h^−1^ and half-lives of 53.3–91.8 h [117,119]. More rapid toxicant elimination characterized by shorter half-lives and larger elimination constants is observed in more resistant animal species. Intraperitoneal administration of AFB1 in pregnant mice at 20 mg/kg led to a serum half-life of 0.3 h in the first 90 min and an elimination constant of 3.0 µg/min [118], while a plasma half-life of 15.5 h was observed in cows orally exposed to AFB1 [89].

AFB1 excretion is most prominent via biliary, urinary, and milk pathways in that order [35,90,103]. More than 2-fold AFB1 excretion occurs through the biliary system compared to urine, the major biliary metabolites being AFB1-Glutathione (AFB1-GSH) and AFP1-Glucuronide; about 10–20% is excreted in the urine in the first 24 h [103]. A review by these authoors shows that the main urinary metabolites in humans and rats are AFM1, AFB1-N^7^-guanine (AFB1-N7-Gua), and AFP1, the major one for these species and monkeys being AFM1, while mice are the only species known to urinary excrete free AFQ1. Indeed, a recent review notes that urinary AFQ1 is rare in both humans and animals [96]. Also, urinary intact AFM1 has been detected in humans [90]. AFB1-N7-Gua, a pro-mutagenic adduct, is removed by nucleotide (NER) and base excision repair (BER) pathways and excreted in urine [4,106]. Alongside these, urinary thiol metabolites, AFB1-GSH, and mercapturic acid (N-acetylcystein or AFB1-Cys-Gly), are detected in humans and animals [35,96,106,115]. Urinary AFB1-dialcohol has also been detected in humans and animals [41]. Glucuronide and/or sulfate conjugates of aflatoxins M_1_, P_1_, Q_1_, B_2a_, and aflatoxicol are excreted in urine and feces [96,97,110,120]. The AFB1 metabolite excreted in the milk of food animals is mainly AFM1, but trace levels of AFQ1, AFM4, AFB2, AFP1, AFG1, AFL M1, and AFL H1 have also been reported [72,83,88,89,96,103]. In poultry, excretion of AFB1, AFM1, AFB2a, and AFL in eggs has been reported [31,32,33,82,95].

The last line of body protection against a toxicant is the expulsion of its phase II metabolites out of target cells. Active translocation of some xenobiotics through cell membranes involving transporters has been described [97,104]. For AFB1, active transport of AFB1-GSH out of cells by two ATP-dependent efflux pumps, namely P-glycoprotein and glutathione S-conjugate carrier [41,110], and extracellular traps (ETs) formation in macrophages that degrade AFB1 via an OS-induced mechanism have been proposed. Lastly, data show a correlation between sensitivity to a toxicant and its excretion kinetics. Animal resistance to the carcinogenicity of AFB1 is negatively correlated with the conversion efficiency of AFB1 to AFP1 and water-soluble metabolites [103,121]. The level of urinary excretion of thiol metabolites (AFB1 mercapturate, together with sulfate and glucuronide conjugates, etc.) is higher in species that are more resistant to the carcinogenicity of AFB1 [35,103].

### 3.5. Toxicodynamics

**Figure 3:**
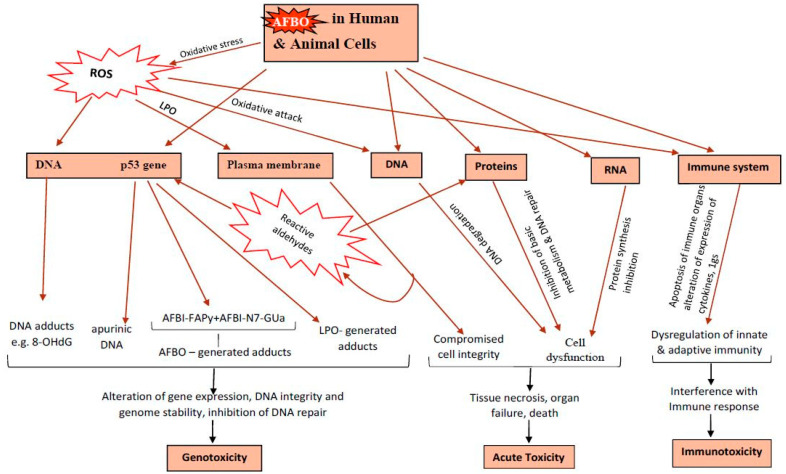
Illustration of toxicodynamic events of AFB1 poisoning and its outcomes in animals and humans. Bio-activation to highly reactive metabolites and oxidative stress are the twin modes of action responsible for AFB1-mediated insults on macromolecules, leading to genotoxicity, acute toxicity, and immunotoxicity.

All mechanisms by which AFB1 exerts its effects are not well understood [114,122]. There are two known modes of aflatoxin poisoning, both involving genotoxicity, immunotoxicity, and acute poisoning by targeting functional macromolecules and immunocompetent cells (Figure 3) [4]. One mechanism is bio-activation by CYP450 enzymes to AFB1-exo-8,9 epoxide (AFBO), an electrophile that insults cellular nucleophiles (nucleic acids, phospholipids, and proteins), inducing cellular dysfunction [123,124,125]. Briefly, the carbon double bond at position 8,9 potentiates AFB1 for bio-transformation to highly electrophilic AFBO [98,122,126], which binds DNA by alkylation targeting the N7 position of the guanine base [4,35,110,127,128] to form 8,9-dihydro-8-(N7-guanyl)-9-hydroxy-AFB1 (AFB1-N7-Gua), a pro-mutagenic lesion [114,129]. AFBO-generated DNA adducts predominantly AFB1-N7-Gua, and the more stable ring-opened AFB_1_-formamidopyrimidine (AFB1-FAPY) and 2,3-dihydro-2-(N-formyl-2,3,6-triamino-4-oxopyrimidine-N-yl)-3-hydroxy AFB1 (AFB1 III) cause missense G-to-T (AGG to AGT) point transversion mutation in the 3rd nucleotide of codon 249 of the p53 gene [110,114,130,131]. This causes the substitution of amino acid arginine with serine, modifying the functionality of the mutant gene product, which promotes the development of hepatocellular carcinoma, HCC [93,113,114,116,132,133,134]. AFB1-FAPY and AFB1 III are more stable and refractory to NER [4,110], making BER vital for their removal, but are not excreted in biofluids [96,129].

Aflatoxin-mediated OS is the other mechanism [135] involving AFBO-initiated formation of reactive species (RS) capable of oxidizing DNA bases [85,122,136], lipids [4,137,138], and proteins [129,139,140]. AFBO-generated ROS are superoxide anion (O_2_-, the primary ROS), hydrogen peroxide (H_2_O_2_) resulting from dismutation of O_2_-, hydroxyl free radicals (^.^OH) via Fenton reaction involving H_2_O_2_, and hydroperoxyl radical [137]. The most common reactive nitrogen species (RNS) is nitric oxide (NO) [141]. Aflatoxins initiate excessive generation of free radicals, usually ROS or RNS, subsequently inducing oxidative or nitrosative stress, respectively, when there is a homeostatic imbalance between pro-oxidants’ (free radicals) levels and the antioxidant system’s ability to detoxify them [4,138,141,142]. Direct insults by free radicals or downstream breakdown of toxic molecules lead to various pathological sequelae such as cellular damage, altered gene expression, and, ultimately, disease [137,143]. AFB1-induced elevated ROS production causes an oxidative attack on lipids, nucleic acids, and proteins, altering their cellular functions and inducing pathological lesions such as lipid peroxidation (LPO), oxidative DNA damage, modification of the antioxidant defense system, and immunosuppression [4,136,144]. Proposed mechanisms of aflatoxin-mediated immunosuppression include increased expression of pro-apoptosis proteins caspases and *Bax* and decreased expression of anti-apoptosis protein *Bcl-2* triggered by increased OS [145,146,147], leading to apoptosis of lymphoid organs and reduced production of humoral factors [148,149]. Another possible pathway is OS-mediated blocking of protein synthesis, inhibition of macrophage migration, blocking of complement hemolytic activity, reduced lymphocyte proliferation through insults on lymphoid organs, and impaired lymphocytic cytokine production [138,150,151,152].

In aflatoxicosis, OS is characterized by reduced activities of antioxidative enzymes such as glutathione peroxidase (GPX), glutathione reductase, catalase (CAT), total superoxide dismutase (SOD), depletion of intracellular antioxidants (glutathione, GSH), and increased malondialdehyde (MDA) in broiler chicken [146,153], rats [85,141,154], and mice [153]. There are elevated levels of reactive species (RS), such as NO, MDA, and ROS in rats [85,141], H_2_O_2_, MDA in mice [153], and MDA in broiler chickens [146,155]. Further, OS-associated apoptosis occurs in hepatocytes [156] and splenic lymphocytes, indicative of AFB1-mediated immunosuppression in broiler chickens [146,155] and testicular cells of sheep [156]. Interestingly, AFB1 also induces ROS-mediated autophagy [85,142]. Dai et al. [85] identified excess ROS production, DNA injury, OS, LPO, apoptosis, mitochondrial dysfunction, autophagy, necrosis, and inflammatory response as the molecular mechanisms implicated in the pathways of AFB1-induced cytotoxicity and cell death.

Oxidative attack on lipids containing carbon double bonds, mostly polyunsaturated fatty acids (PUFAs) in cell membranes by free radicals, leads to LPO and generation of breakdown reactive aldehydes, namely 4-hydroxy-2-nonenal (4-HNE) and MDA [136,137]. Ayala et al. [138] noted that the production of free radicals is triggered by exogenous stimuli such as environmental toxins to initiate the LPO process, a chain reaction generating MDA and 4-HNE, which attack proteins and DNA. MDA and 4-HNE adducts are involved in cellular processes and have injurious effects, including protein/DNA crosslinking, which causes modification of biochemical properties of biomolecules, resulting in pathological lesions [138]. AFB1-generated RS, including downstream LPO-generated MDA and 4-HNE, are capable of inducing protein damage by oxidizing side chain amino acids such as lysine, arginine, and threonine to yield carbonyl derivatives and protein carbonyl [136,139,140]. The molecular mechanism for AFB1-mediated growth reduction in animals and humans is not known [53]. One proposed pathway is apoptosis due to AFB1-driven DNA damage-blocking growth, as was observed in nematodes [157]. Loss of enzyme function is another possible mechanism, where protein synthesis is impaired through structural modification by crosslinking of MDA and 4-HNE with elongation factor-2, which catalyzes ribosome translocation along m-RNA during the elongation phase of translation [138,158].

Hydroxyl radicals initiate oxidative DNA insult, inducing DNA mutation, cell apoptosis, replication aberrations, and genomic instability [137]. The major molecular lesion of oxidative DNA damage is 8-hydroxydeoxyguanosine (8-OHdG), another mutagenic adduct resulting from insults of DNA-guanine by OS-generated hydroxyl radical [4]. 8-OHdG and AFBO are involved in the initiation stage of carcinogenesis [159]. Unlike AFBO-generated DNA adducts, 8-OHdG does not specifically target the p53 gene [4]. It is responsible for OS-mediated genotoxicity associated with AFB1, triggering gene mutation through base modification by mispairing with adenine during DNA replication, initiating G–C to T–A transversion point mutations prevalent in mutated oncogenes and tumor suppressor genes [4,137,144]. It is a potent genotoxin with mutagenic effects in mammals, bacteria [137], and birds [136]. To minimize 8-OHdG accumulation within the genome, antioxidants and free radical-scavenging enzymes, notably the BER, are the defense and repair mechanisms that counter its effects [4,144].

### 3.6. Toxicity

Toxicological outcomes of dietary aflatoxins are acute toxicity, sometimes resulting in death, and various chronic manifestations, depending on microsomal activity, species, breed, age, sex, nutrition, environmental stress, concomitant exposures, dose, and duration of exposure [39,94,148,160,161]. Genotoxicity, mutagenicity, and carcinogenicity of AFB1 are attributed to DNA damage by AFBO [4,35,114,127] and ROS [92,136], as detailed in Section 3.5. AFB1 has a wide tissue tropism, its main target organ being the liver, and is a potent hepatotoxin and hepatocarcinogen in man and many animal species [5,159,162,163]. Acute aflatoxicosis is characterized by acute hepatitis, jaundice, hepatosplenomegaly, lethargy, depression, nausea, anorexia, ascites, leg edema, febrile episodes, tachycardia, fatty infiltration (hepatic lipidosis), hemorrhagic and centrilobular necrosis of the liver, bile duct hyperplasia/proliferation, aflatoxin residues, AFB1-protein adducts, notably lysine adducts, and a high mortality rate [4,5,10,55,94,95]. Effects of chronic aflatoxicosis are generally mutagenicity and carcinogenicity, reduced animal productivity [94,164], growth faltering, immunomodulation [53,158,161], broad dysfunctions in GIT [165,166], damage of several organs [5,94,97,167], and reproductive defects [4,94,168]. Although CYP450 enzymes are found in almost all cells, their levels in different tissues vary significantly [97]. This explains the observed differences in the risk of aflatoxin poisoning for various organs, such as the liver, kidneys, small intestines, and lungs [4,97].

#### 3.6.1. Toxicity in Humans

Naturally occurring aflatoxins are classified as Group 1 carcinogens, that is, potently carcinogenic to humans [35]. Of these, AFB1 is the most potent mutagen, followed by AFG1, while AFB2 and AFG2 are non-mutagenic [35,127]. Their metabolites, AFL, AFM1, AFLH1, and AFQ1, have increasing mutagenic activity in that order, while AFP1 and AFB_2a_ are non-mutagenic. AFBO-generated (AFB1-N7-Gua, AFB1-FAPY, and AFB1 III) and OS-associated (8-OHdG) DNA adducts confer epigenetic changes via corruption of specifically tumor suppressor p53 protein (TP53 or Guardian of the Genome), or various oncogenes and tumor suppressor genes, respectively [4,114]. TP53 is a transcription factor encoded by the p53 gene that regulates cell survival, apoptosis, senescence, and DNA repair [169,170]. Its inactivation by transverse point missense mutation in the p53 gene is a hallmark of carcinogenesis, with the mutant p53 protein losing its tumor suppression function or corrupted to promote oncogenesis [130,133,169,170]. Missense mutations are more common in areas with a high prevalence of dietary aflatoxin, chronic hepatitis B virus (HBV) or hepatitis C virus infections, and alcohol consumption [35,114,133,134,169,171].

AFB1 has been linked to cancers, most prominently hepatocellular tumors, in several animals [114,129,160]. In humans, AFB1 is suspected to be associated with cervical [8], lung [158], and esophageal cancers [7] and is strongly linked to HCC [35,114,122]. Using urinary aflatoxin [53,122], a significant positive effect of dietary aflatoxin on HCC development and increased risk of HCC due to interaction between aflatoxin and HBV infection was demonstrated in SSA, including Kenya, SEA, and China, with co-occurrence of HBV infection posing an extremely high risk, especially in high prevalence areas [35,39,54,57,114,172]. This interaction is actually more consistent with additive rather than multiplicative effects [4,6,114,122,172,173]. Indeed, AFB1, on its own, has significant carcinogenic potency in the development of HCC in humans [172,173]. Increased risk of HCC in *GSTM1*-null genotype humans [35] and transgenic mice deficient in *Xeroderma pigmentosum* A, a protein critical for NER of damaged DNA [35,122], suggests genetic polymorphism plays a vital role in aflatoxin-mediated HCC. These data form the basis of analysis of HCC risk in the human population in areas with chronic aflatoxicosis and HBV infection employing a deterministic approach [5,174,175]. The risk is a product of AFB1’s carcinogenic potency (a function of seropositive and seronegative individuals for the surface antigen of HBV) and estimated daily intake per body weight [88]. There is a dearth of comprehensive updated risk analyses for HCC associated with dietary aflatoxin in maize, dairy products, and other common staple foods.

An association between aflatoxin intake and growth retardation in humans, a condition termed childhood stunting or growth faltering [53,114,158,176] has been reported in Kenya [6], Tanzania [63], and the Gambia [177]. Because of their lower body weight, higher rate of metabolism, and inferior toxin detoxification capacity, children are more susceptible to aflatoxin poisoning than adults [63]. Indeed, chronic exposure to high AFB1 levels at infancy in Gambian children was associated with growth faltering, an effect that was more pronounced when exposure commenced in utero [177]. This condition, which causes early life morbidity and mortality, requires effective intervention and mitigation strategies to protect in utero, infantile, and pediatric individuals from aflatoxin poisoning. It is, however, possible that, due to confounding factors, the association between aflatoxin exposure and growth in children and in utero could be overestimated in the observation research above. A study employing a randomized control trial design to eliminate this shortfall showed no causal link between child linear growth and dietary aflatoxin exposure in Kenya [178]. They further observed age-specific effects that need further research. Cohort studies also did not show any relationship between aflatoxin exposure and child growth in Nepal [179] and in Bangladesh [180].

#### 3.6.2. Toxicity in Animals

Aflatoxicosis in domestic animals is characterized by general unthriftiness, anorexia, GIT problems, reduced feed utilization efficiency, and mortality, among other problems [95,125,160,161,163]. Impaired productivity in terms of growth rate and feed conversion efficiency in broiler chicken [161,181,182] and pigs [183], egg production and hatchability in layer chicken, and milk production in dairy cattle have been observed in aflatoxin-exposed animals [22,82,94,125,184] resulting from aflatoxin-induced multiple organ dysfunction [37]. This is preceded by inappetence [94,161,182] due to toxicity [164]. In addition, AFB1 induces nutrient malabsorption and metabolism aberrations [94,166,168,181]. Dietary aflatoxin also adversely affects the quality or characteristics of edible animal products. These include toxic residues in milk, eggs, and meat, poor eggshell quality in layer poultry, and bruising in broiler carcasses [27,34,37,82,95,160,185,186]. Generally, the performance response to aflatoxin exposure is a reduction in both feed intake and growth rate in pigs and broiler chickens [161,183].

#### 3.6.3. Immunotoxicity in Humans and Animals

Various components of the immune system exhibit differential responses to aflatoxin poisoning. Cellular responses and non-specific humoral factors such as complement and interferon are impaired by relatively lower levels of aflatoxin [160]. On the other hand, T-lymphocytes are more sensitive than B-cells [187]. AFB1 inhibition effects on cell-mediated immunity (CMI) include thymic aplasia, inhibition of macrophage phagocytosis, delayed cutaneous hypersensitivity, reduced delayed-type hypersensitivity, and cutaneous basophil hypersensitivity (CBH), suppressed graft-versus-host response, reduced lymphoblastogenesis, delayed and reduced lymphocyte proliferation, and leukocyte migration [151,160,187,188]. At the organ level, AFB1 induces CMI suppression characterized by thymus atrophy and aplasia with reduced percentages of mature thymocytes, thymus architectural loss, and increased apoptotic thymocytes in broiler chickens [147]. AFB1 depresses adaptive and innate components of the immune system [4,82,189]. An inhibited immune cell proliferation index characterized by delayed and reduced lymphocyte proliferation in response to vaccine antigen and a depressed percentage of specialized T-cell subsets indicates impaired lymphocyte activation in AFB1-exposed humans, poultry such as broiler chickens, and other animals [152,187]. These immunosuppressive effects could lead to poor vaccine response, reduced therapeutic efficacy, increased susceptibility to infections [4,158,190] in humans and animals, and aggravate disease pathogenesis [191,192,193,194]. Indeed, experimental chronic exposure to AFB1 impaired vaccine response in pigs [188], and poultry, including chicken [82,189,195].

Concerning humoral immunity (HI), AFB1 induces immunosuppression characterized by atrophy, disruption of normal architecture, and functional impairment of immune-competent organs such as the bursa of Fabricius, spleen, and thymus in broilers [30,147,148,196,197,198,199]. The bursa of Fabricius, responsible for the maturation of B and memory cells and therefore important for antibody production and adaptive immunity, is generally a major target of aflatoxins in poultry [197]. In addition to experimentally inducing atrophy of the bursa of Fabricius in broiler chickens [196] and of the spleen in layer chickens [200], AFB1 also triggers massive apoptosis of splenic lymphocytes in broiler chickens [146]. This immunosuppression may lead to decreased antibody titers and reduced vaccine efficacy [30,82,151,189,198,201,202,203]. In humans naturally exposed to dietary aflatoxin, lowered, reduced salivary immunoglobulin A secretion was observed [158]. Up-regulation of pro-inflammatory (IL-6, IFN-γ) and regulatory (IL-10) cytokines and inhibition of complement activity are remarkable effects of aflatoxins on non-specific humoral factors [150,152,160]. However, aflatoxins induce a hormetic response exhibiting dose-dependent biphasic effects on HI in chickens and other animals. This is particularly their effect on the alternative pathways of complement activation, characterized by low-dose stimulation and high-dose inhibition depending on the level and duration of exposure [114,152,187,189,204].

### 3.7. Detection of Dietary Aflatoxin

Measurement of dietary aflatoxin is a 3-step process that constitutes the mycotoxin sampling plan: sample selection, its preparation, and toxin detection/quantification [22,23,205,206,207]. These steps are sampling stages, which are critical for accurate and precise measurement necessary for true and reliable estimation of the characteristic [208,209,210]. Chromatographic methods, namely thin-layer chromatography (TLC), liquid chromatography (LC), gas chromatography, and their improved versions such as high-performance (HP) TLC (HPTLC), HPLC, ultra HPLC (UHPLC), LC-mass spectrometry (MS) (LC-MS), LC tandem MS (LC-MS/MS), and immunochemical methods/immunoassays are employed for detection and quantification [40,54,66,75,79,211,212]. Other methods are spectroscopy and emerging approaches based on hyperspectral imaging, aptamers, fluorescence/near-infrared spectroscopy, molecularly imprinted polymers (MIPs), surface plasmon resonance detection, optical waveguide light-mode spectroscopy, nanotechnology, and acetylcholinesterase inhibition [6,22,40,54,92,94,212,213]. Indeed, molecular imprinting technology-enhanced solid phase extraction is a promising state-of-the-art sample clean-up approach that will drastically reduce the matrix effect during the analysis of real samples for dietary mycotoxins [214].

Immunoassays apply antigen–antibody reaction binding specificity to detect aflatoxin and include enzyme-linked immunosorbent assays (ELISAs), radioimmunoassay, lateral flow immunoassays (immunodipsticks), and immunoaffinity fluorometry [54,64,65]. Others are fluorescence polarization immunoassay, biosensors, and biosensor-based devices comprising the mycotoxin-specific antibody and a transducing element (enzyme, peptide, aptamer, or MIPs) that converts the change in physical variable produced by the antibody-mycotoxin reaction into a measurable signal [22,54,212,213]. Due to superior sensitivity, specificity, rapidity, simplicity, cost-effectiveness, and high sample throughput with low sample volume, the application of immunoassays is widespread and accepted for the quantification of dietary aflatoxins [6,40,92]. Competitive ELISA (cELISA), the predominant method and a preferable tool when the antigen is a hapten with a single epitope, has two variants available for aflatoxin analysis [22,215]. Direct inhibition cELISA has been employed to determine aflatoxin in animal feed and human foods, including milk [64,65,66,70,71,72,73,216], while Mutegi et al. [67] applied indirect cELISA to quantify AFB1 in peanut products. Highly sensitive nanotechnology-based immunoassays such as cELISAs have been developed [40,54,212,217].

Aflatoxin analysis, however, starts with sample selection followed by sample preparation until a test portion is acquired, which is then subjected to pre-treatments such as clean-up and concentration manipulations prior to toxin detection and quantification [22,206]. Sample selection involves the identification of a sampling unit from which incremental samples are selected by random collection of an adequate number and size from various localities and pooled to give an aggregate sample [75,206,218,219]. Sampling tools and procedures are sources of bias that can violate the requirement of an equal chance of being selected [220]. Sample preparation entails representative mass reduction of the aggregate sample to laboratory sample [22] through dry milling, random reduction [16,19,206,221], and then random identification of the test portion, which is further homogenized by wet milling/water slurries [15,210,219]. To minimize matrix effects and enrich extract, the sample undergoes pre-treatment [22], commonly solvent extraction and clean-up solid-phase extraction (SPE) [92,94,221]. In classical SPE, the solvent removes the analyte from the sample, facilitated by agitation, then spinning or filtration before enrichment and clean-up [22]. Aflatoxin is separated from other unwanted interfering materials based on relative solubility by selectively moving from the aqueous to the polar organic component of the solvent mixture [40]. Several systems have been developed for sample clean-up to disengage interfering substances prior to mycotoxin quantification by advanced equipment such as HPLC. These are SPE (octadecylsilane/C18, immuno-affinity, and multifunctional columns, MIPs), instrumental solvent extraction systems (microwave-assisted and ultrasonic products), and extraction-clean-up–concentration combination products (QuEChERS, matrix solid-phase dispersion, dispersive liquid–liquid micro-extraction) [40,92,94]. Quick, easy, cheap, effective, rugged, and safe (QuEChERS) system is a multi-mycotoxin SPE system.

The largest uncertainty associated with the measurement of aflatoxin content is due to its heterogeneity, leading to variability [17,22,219]. It is difficult to acquire a representative sample that accurately estimates true aflatoxin content [21,23]. Development of improved analytical methods often focuses on downstream steps, yet the sample selection step is the largest source of variability, followed by sample preparation, while quantification is the smallest contributor [6,13,23,205,206,219]. There is a need for a reliable test procedure with improved accuracy and precision for the estimation of true aflatoxin content in chicken feed [25]. Recent data on aflatoxin contamination in figs [219] and maize [16] suggest that optimization of upstream procedures can considerably reduce measurement uncertainty. For instance, optimizing sample selection procedures and then incorporating a wet milling (water slurring) step in the sample preparation procedure, a more efficient comminution procedure than dry milling, is a critical modification [13,15,16,17,206,210,218,222].

### 3.8. Management of Dietary Aflatoxicosis

Aflatoxin management requires a multi-faceted approach employing various intervention/mitigation strategies, the most promising being chemoprotection and enterosorption. The different strategies employed for the management of dietary mycotoxins are given in Table 1. Chemoprotection by yeast extracts, pharmaceutical and phytochemical products, vitamins, and trace minerals involves modulation of toxin activation and detoxification pathways, while enterosorption entails selective, effective adsorption and sequestration of dietary hazards blocking their bioavailability in the GIT, a mechanism utilized by clay aflatoxin binders [6,158]. Legislative control involves institutional regulation where thresholds of dietary mycotoxins are established and stipulated in regulatory standards, and compliance is enforced by official entities [223]. These include FAO/WHO’s *Codex Alimentarius*, the Kenya Bureau of Standards, KEBS [224,225], the US Food and Drug Administration, FDA [226], the European Union, EU [227], and the East African Community, EAC. In developing countries, aflatoxin levels above legal limits are common [228,229], indicating low-risk awareness, a lack of enforcement of regulatory limits, and an official nonchalant attitude [53,230]. Currently, there is a need for a comprehensive review update of the national and regional human food and animal feed contaminants regulatory systems in relation to dietary mycotoxin as a food safety hazard in the SSA.

Other aflatoxin mitigation strategies include good agricultural practice [5,6,86,230], nutritional supplementation with nutraceuticals [42,149,155,241,242], and physical methods such as irradiation and plasma technology [54,91,95,126]. Where food items comprised of heat-sensitive nutrients, for instance, fruit materials, are involved, traditional non-thermal techniques such as cold plasma technology, irradiation with UV light or γ-rays, and ozone treatment are more desirable for aflatoxin detoxification [54,91,126]. These methods suppress toxigenic *Aspergillus* spp. and degrade the aflatoxin molecule. Plasma technology, an electrical energization of gaseous matter at various levels of atmospheric pressure, is an upcoming practical, cost-effective decontamination approach and a suitable alternative to thermal-based methods [91]. A promising biocontrol technology for dietary aflatoxins involves adjustment of soil microbiome through the application of genetically modified atoxigenic fungal antagonist strains, which displace toxigenic strains from ecological niches by biocompetitive exclusion [5,54,69,95,149,212,231]. Many aflatoxin biopesticidal products, such as Afla-guardR, AF36R, and Aflasafe, have been developed and registered in many countries [5,86]. Other biocontrol agents are probiotics [18,54,243,244] and probiotics with toxin-degrading enzymes [42]. Treatment with chemicals, including nixtamalization, is another effective control strategy [54,70,91,212,256]. Novel strategies under experimentation are vaccination [211], predictive technology [223], antidote development, and nano-based mold inhibitors [42].

Nutraceutical agents such as dietary vitamins (A, C, and E) and essential trace minerals (zinc, selenium, and functional amino acids) play protective roles as antioxidants [42,149,155,241,242]. Amelioration of aflatoxicosis effects through modulating xenobiotic metabolism using chemo-protective pharmaceuticals and phytochemical agents is an active area of research. Pharmaceuticals include aflatoxin-blocking agents modulating both phase I and II reactions, notably oltipraz, butylated hydroxytoluene, ethoxyquin, indole-3-carbinol, and phenethyl isothiocyanate [35,232,233,234]. Phytochemicals act as free radical scavengers against aflatoxin poisoning [125]. They include chlorophyllin [35,235], luteolin [154], flavonoids [238], kolaviron, a natural biflavonoid [236], ß-caryophyllene [239], curcumin, a natural polyphenol [85,149,153,257], ferulic acid [240], and lycopene, a carotenoid pigment [136,237]. The development of effective adsorbents/binders, biodegrading enzymes, and probiotics to ameliorate mycotoxin effects by remediation or GIT detoxification is ongoing [42,69]. In-feed anti-mycotoxin additives (AMAs) target mycotoxins by adsorbing, binding, or detoxifying by bio-transformation are available, but there are many products marketed as AMAs whose efficacy is largely unknown [258]. Indeed, many AMAs are ineffective [259]. A good AMA effectively sequestrates mycotoxin(s), ameliorates toxin’s injurious effects, is non-toxic (free from heavy metals and dioxins), cost-effective, has no adverse effects on edible animal products, does not mask mycotoxins, is stable to feed processing procedures, and its use and efficacy must be verifiable [231,260]. It has high and broad-spectrum adsorption capacity and binds mycotoxins selectively and irreversibly at different pH levels. In addition, it does not interfere with nutrients and therapeutic drugs [247,248].

Mycotoxin binders are broadly categorized as inorganic (mineral clays, activated charcoal), organic, mixed inorganic/organic AMAs, and synthetic polymers. Clay minerals are a diverse group of aluminosilicates classified according to their structure, comprising mainly hydrated aluminosilicates (hydrated sodium calcium aluminosilicates: HSCAS), phyllosilicates (montmorillonite, smectites, and beidellite), and tectosilicates (zeolite/clinoptilolite) [54,148,231,249]. HSCASs, such as Novasil Plus, have high cation exchange capacity and selectively immobilize aflatoxin by chemisorption through the complex formation by the ß-keto-lactone or bilactone moieties of aflatoxin and metal ions of HSCAS during digestion, therefore blocking its bioavailability from the GIT [245,246,247,248]. Synthetic polymers, such as cholestyramine and polyvinylpyrrolidone, are also used as aflatoxin binders [54,231,252]. Other inorganic mineral binders are bentonite, activated charcoal from pyrolysis of organic materials, kaolin, diatomaceous earth [30,148,231,247], and a novel magnetic reduced graphene oxide composite, a nano-adsorbent with high adsorption efficiency for AFB1 [261]. The limitations of clays are that they are non-biodegradable and often accumulate in manure, contaminated with toxic metals and dioxins, and therefore, they necessitate rigorous testing before use. They also have a narrow binding spectrum in favor of aflatoxins [247,262,263]. Some also adsorb trace nutrients [249]. The development of organic binders such as polymeric organic polysaccharide-based adsorbents addresses these drawbacks [264,265]. In Kenya, chicken manure is used by dairy and crop producers [266], making organic binders more environmentally friendly. Additionally, yeast-based binders such as mannanoligosaccharides have nutritional [250,252] and immunomodulatory values [201]. There is a dearth of information on the effectiveness of locally and commercially available feed additive products imported and touted as “mycotoxin binders” [5,37,258]. In addition to this uncertainty and despite their widespread use, these products are neither governed by food safety standards nor can they be detected analytically, making their regulation very difficult. Rigorous and continuous evaluation of these products to ascertain their efficacy as ameliorating agents against the effects of animal aflatoxicosis is necessary [69,259].

Common organic binders are humic acid, yeast (*Saccharomyces cerevisiae*), polysaccharide-based adsorbents mainly yeast cell-wall-based mannan, glucan, plant-derived glucomannan and glucan, mannanoligosaccharides, esterified glucomannan (EGM), lactic acid bacteria, and hybrids of inorganic–organic AMA products [37,149,188,231,247,250,252]. These products, especially yeast cell-wall-based adsorbents, exhibit different absorption mechanisms, such as hydrogen bonding and ionic or hydrophobic interaction for sequestering mycotoxins [248,252]. The cell wall of *S. cerevisiae* is a complex structure with many components [252,255,267], of which β-D glucans provide accessible binding sites for sequestrating mycotoxins [251,252,253,254], whose binding efficacy depends on both its molecular structure and of mycotoxins [268].

The amelioration activity of AMA has been evaluated (Table 2). In vitro binding efficacy of activated charcoal, sodium and calcium bentonite, and EGM products for AFB1 [255,269,270], yeast-based products for AFB1 and ZEA [247], EGM for AFB1, OTA, and T-2 toxin [201], and of various clays for OTA and ZEA [271] was demonstrated at pH values of GIT. An in vitro study demonstrated the high binding capacity of NovaSil Plus^®^ and AFB1 with no interaction with vitamin A [272]. Yeast and plant-based adsorbents, including glucomannan, protect animals against mycotoxicosis [54,188]. Glucomannan is protective against aflatoxicosis in merino rams [273] and horses [231], while Novasil Plus^®^ reduced AFM1 levels in cattle milk [274]. In humans, in situ, detoxification potential of bentonite clay binder and fumonisin esterase [275] and effective, safe reduction in bioavailability of dietary aflatoxins by NovaSil, a calcium montmorillonite clay, were demonstrated [276,277]. However, it has been proposed that aflatoxin-binding technology is not ethically acceptable for humans due to food safety concerns [278]. Precisely, this will conflict with established food safety infrastructure that discourages consumption of aflatoxin-contaminated food.

In poultry, dietary glucomannan showed a protective effect against aflatoxicosis in broiler chicken [201,251,279,280] and quails [281], while glucan protected layer chicken [28]. In broiler chicken, activated charcoal and bentonite reduced aflatoxicosis-induced hepatic lesions, restored a number of immune cells, and improved their general performance, with the most effective binder being bentonite [30,282]. Mycosorb^®^ restored aflatoxin-altered feed conversion efficiency [80]. Dietary bentonite reduced liver AFB1 residues by half [186], and NovaSil Plus^®^ counteracted aflatoxin-induced serum biochemical lesions, altered organ weights [163], and generally alleviated the effects of aflatoxicosis in broiler chicken [279,283,284]. Similarly, a hybrid AMA yeast (*S. cerevisiae*)-zeolite ameliorated the impact of aflatoxicosis in broiler chicken [250,285]. Meta-analysis of anti-aflatoxin additives in poultry feed showed that inorganic binders are more protective, followed by antioxidants, and organic binders in that order [248]. Novasil Plus^®^ (phyllosilicate clay: calcium montmorillonite, BASF^®^), Myosorb A+^®^ (*Saccharomyces cerevisiae* yeast cell wall extract, HSCAS, algae oil; Alltech^®^ Inc.), and Mycofix Select 3.0^®^ (synergistic adsorbent minerals; bio-degrading enzyme: FUMzyme-producing *Coriobacteriaceae* bacterium: BBSH; bio-protective phytochemicals: plant and algae extracts; Biomin^®^) are among the nine commercially available AMAs in Kenya that need evaluation [258]. Modern broiler breeds have a more efficient nutrient conversion system, requiring faster hepatic metabolism [189]. This makes them more susceptible to the effects of AFB1 due to up-scaled xenobiotic metabolism. Continuous evaluation of the effects of dietary AFB1 on the performance of emerging new broiler breeds is, therefore, necessary. Identification of an appropriate broad-spectrum dietary AMA against aflatoxicosis is a high priority.

## 4. Conclusions

This review highlights that in spite of all the research that has been conducted since aflatoxins were discovered in the 1960s, many issues relating to them and the other mycotoxins are still not well understood. The functional significance of mycotoxins in fungal biology is still under investigation, with some potential roles being enhancing ecological niche advantage, anti-fungivore activity, facilitating host invasion, and oxidative stress adaptation. In food toxicology, mycotoxins, especially aflatoxins, are important dietary toxicants. Toxicodynamics of AFB1 primarily involves two mechanisms of toxicity: bio-activation and oxidative stress (OS). However, the biochemistry of aflatoxin poisoning is still not fully understood. Dietary aflatoxin induces significant toxicity implicated in both acute and chronic health conditions in humans and animals. Acute aflatoxicosis prominently causes extensive liver damage, while chronic exposure induces genotoxicity/mutagenicity, carcinogenicity, and immunosuppression. In food animals, chronic aflatoxicosis causes poor animal performance and introduces violative residues in edible animal products. The ubiquity of mycotoxins, as well as the lack of/or inadequate enforcement of regulations in many countries, means that the health burdens associated with mycotoxins are likely to persist. While it was not possible to list all studies on the occurrence of various mycotoxins in this review, it seems evident that their prevalence is very high, and what is described in the literature is probably just the tip of the iceberg.

Effective mitigation strategies depend on reliable detection and management methodologies of the dietary hazard. There are still pending issues relating to the optimization of aflatoxin residue estimation methods. Despite having a trend towards more advanced, specific, and sensitive analytical methods with enhanced detection capabilities, more attention should be paid to representative selection and preparation of samples, which is crucial for reliable measurement of dietary aflatoxins. Management of dietary aflatoxins is multi-faceted, employing different intervention strategies and a holistic approach integrating chemoprotection, enterosorption, good agricultural practices, and regulatory and biocontrol measures. Chemoprotection based on modification of xenobiotic metabolism and enterosorption (using aflatoxin binders), which ameliorate the effects of aflatoxin by altering the hazard’s toxicokinetics, are the most applied approaches. Nevertheless, effective mitigation strategies are yet to be established. For the time being, surveillance employing predictive technology and mitigation strategies employing biocontrol agents such as aflatoxin bio-pesticides, probiotics, or probiotics with toxin-degrading enzymes, and aflatoxin sequestration using binders seem to be promising approaches.

## Figures and Tables

**Table 1:** Various management strategies against aflatoxin poisoning.

Mitigation Strategy/Product	References
Good agricultural practice (GAP)	[5,6,86,95,231]
Legislative control: Institutional regulation	[53,223,224,225,226,227,228,230]
Physical methods: Sorting, heat treatment, hermetic storage bags, irradiation, and cold plasma technology	[54,91,95,126,231]
Traditional chemical methods	
Treatment with chemicals: Salts, acidic/alkaline compounds, ozone, and chitosan nanoparticles	[54,212]
Nixtamalization	[70,91]
Chemo-protection (xenobiotic metabolism modulation)	
Pharmaceuticals: oltipraz, butylated hydroxytoluene, ethoxyquin, indole-3- carbinol, and phenethyl isothiocyanate	[35,232,233,234]
Phytochemicals: beta-caryophyllene, chlorophyllin, curcumin, ferulic acid, flavonoids, kolaviron (a natural biflavonoid), luteolin, and lycopene	[35,85,125,136,149,153,154,235,236,237,238,239,240]
Nutraceuticals: Dietary vitamins (A, C, and E) and essential trace minerals (zinc, selenium, and functional amino acids)	[42,149,155,241,242]
Biocontrol	
Probiotics: bacteria, yeast species, mannan-oligosaccharides, and combination of probiotics with toxin-degrading enzyme	[42,149]
Experimental probiotics*: Bacillus*, *Lactobacillus*, *Saccharomyces*, *Trichoderma, Penicillium, Pseudomonas, Ralstonia, Burkholderia, Streptomyces, Stenotrophomonas*, *Bacillus*, *Serratia*, *Pediococcus,* and *Lactococcus*	[18,54,212,243,244]
Aflatoxin biopesticides: Afla-guardR^®^, AF36R^®^, Aflasafe^®^	[5,54,69,86,95,149,212,231]
Gastrointestinal detoxification using dietary AMAs	
Inorganic AMAs: Aluminosilicates: hydrated aluminosilicates (HSCAS, e.g., Novasil^+^), phyllosilicates (montmorillonite, smectites, and beidellite), and tectosilicates (zeolite/clinoptilolite), bentonite, activated charcoal, kaolin, and diatomaceous earth	[54,148,231,245,246,247,248,249]
Organic AMAs: Humic acid, yeast (*Saccharomyces cerevisiae*), polysaccharide-based adsorbents, mainly yeast cell wall-based mannan, glucans, plant-derived glucomannan and glucan, mannanoligosaccharides, esterified glucomannan, lactic acid bacteria, and hybrids of inorganic–organic AMAs	[37,149,188,231,247,248,250,251,252,253,254,255]
Synthetic AMAs: Cholestyramine, polyvinylpyrrolidone	[54,231,252]
Strategies under experimentation	
Nutritional Strategy: Nutraceuticals such as functional amino acids	[42,149,155,242]
Other novel strategies: Vaccination, predictive modeling, antidote, and nanotechnology-based mold inhibitors	[42,211,223]

**Table 2:** Studies to investigate the efficacy of various AMAs. In situ, in vitro, and in vivo systems have been employed to evaluate the amelioration activity of several anti-mycotoxin products.

Type of Mycotoxin	AMAs Evaluated	Experimental Evaluation System	References
AFB1	Activated charcoal, sodium, calcium bentonite, EGM products	in vitro	[255,269,270]
AFB1, ZEA	Yeast-based products	in vitro	[247]
OTA, T-2 toxin	EGM	in vitro	[201]
OTA, ZEA	Various clays	in vitro	[271]
AFB1	NovaSil Plus	in vitro	[272]
AFB1	Glucomannan	Broiler chicken	[201,251,279,280]
AFB1	Glucomannan	Quails	[281]
AFB1	Glucan	Layer chicken	[28]
AFB1	Activated charcoal, bentonite	Broiler chicken	[282]
AFB1	Mycosorb^®^	Broiler chicken	[80]
AFB1	Bentonite	Broiler chicken	[186]
AFB1	NovaSil Plus	Broiler chicken	[163,279,283,284]
AFB1	Yeast (*S. cerevisiae*)-zeolite	Broiler chicken	[250,285]
AFB1	Glucomannan	Merino rams	[273]
AFB1	Glucomannan	Horses	[231]
AFB1	Novasil Plus	Dairy cows	[274]
AFB1 + FB1	Bentonite clay-fumonisin esterase	Humans (in situ)	[275]
Total aflatoxins	Novasil	Humans (in situ)	[276,277]

Key: EGM = Esterified glucomannan.

## Data Availability

No new data were created or analyzed in this study. Data sharing is not applicable to this article.

## Abbreviations

·OH	hydroxyl free radicals
4-HNE	4-hydroxy-2-nonenal
8-OHdG	8-hydroxydeoxyguanosine
ADME	absorption, distribution, metabolism and excretion
AFAR	aflatoxin aldehyde reductase
AFB1	aflatoxin B_1_
AFB1-GSH	aflatoxin B_1_-glutathione
AFBO	AFB1-exo-8,9 epoxide
AFB1 III	2,3-dihydro-2-(N-formyl-2,3,6-triamino-4-oxopyrimidine-N-yl)-3-hydroxy AFB1
AFB1-Cys-Gly	aflatoxin B1-Cysteine-Glycine adduct
AFB1-dhd	AFB_1_-dihydrodiol
AFB1-FAPY	AFB_1_-formamidopyrimidine
AFB1-N7-Gua	8,9-dihydro-8-(N7-guanyl)-9-hydroxy-AFB_1_
AFB2	aflatoxin B_2_
AFB2a	aflatoxin B_2a_
AFB3	aflatoxin B_3_
AFBO	aFB1-exo-8,9 epoxide
AFG1	aflatoxin G_1_
AFG2	aflatoxin G_2_
AFG2a	aflatoxin G_2a_
AFGM1	aflatoxin M1
AFGM2	aflatoxin GM_2_
AFGM2a	Aflatoxin GM_2a_
AFL	aflatoxicol
AFL H1	aflatoxicol H_1_
AFL M1	aflatoxicol M_1_`
AFM1	aflatoxin M_1_
AFM1-P1	4,9a-dihydroxyaflatoxin B_1_
AFM2	aflatoxin M_2_
AFP1	aflatoxin P_1_
AFQ1	aflatoxin Q_1_
AFQ2a	aflatoxin Q_2a_
AMAs	anti-mycotoxin additives
AUC	area undet the curve
Bax	Bcl-2-associated protein x
Bcl	B-cell leukemia
BER	base excision repair system
C18	octadecyl
CAT	catalase
CBH	cutaneous basophil hypersensitivity
CL	clearance
Cmax	maximum plasma concentration
CMI	cell-mediated immunity
CYP450	cytochrome P450 system
DNA	deoxyribonucleic acid
DON	deoxynivalenol
EAC	East African Community
EGM	esterified glucomannan
EPHX	epoxide hydrolase
ETs	extracellular traps
EU	European Union
ELISA	enzyme-linked immunosorbent assay
cELISA	competitive enzyme-linked immunosorbent assay
FAO	Food and Agriculture Organization of the United Nations
FB1	fumonisin B_1_
FDA	Food and Drugs Administration, United States of America
GIT	gastrointestinal tract
GPX	glutathione peroxidase
GSH	glutathione
GSTs	glutathione-*S*-transferases
H_2_O_2_	hydrogen peroxide
HBV	hepatitis B virus
HCC	hepatocellular carcinoma
HI	humoral immunity
HPLC	high performance liquid chromatography.
HPTLC	high-performance thin layer chromatography
HSCAS	hydrated sodium calcium aluminosilicates
IFN-γ	interferon-gamma
IL-10	interleukin 10
IL-6	interleukin 6
KEBS	Kenya Bureau of Standards
kel	elimination rate constant
LADME	liberation, absorption, distribution, metabolism and excretion
LC	liquid hromatography
LC-MS	liquid chromatography-mass spectrometry
LC-MS/MS	liquid chromatography–tandem mass spectrometry
LPO	lipid peroxidation
MDA	malondialdehyde
MIPs	molecularly imprinted polymers
m-RNA	*messenger ribonucleic acid*
MS	mass spectrometry
NATs	acetyltransferases
NER	nucleotide excision repair system
NO	nitric oxide
O2-,	superoxide anion
OS	oxidative stress
OTA	ochratoxin A
8-OHdG	8-hydroxydeoxyguanosine
p53 gene	protein 53 gene
PHS	prostaglandin H synthase
QuEChERS	quick, easy, cheap, effective, rugged, and safe
RNS	reactive nitrogen species
ROS	reactive oxygen species
SEA	South-East Asia
SOD	superoxide dismutase
SPE	solid-phase extraction
SSA	sub-Saharan Africa
SULTs	sulfotransferases
TLC	thin-layer chromatography
Tmax	time it takes for a toxin to attain maximum concentration in circulation
UDP	uridine 5′ diphosphate
UGTs	uridine 5′ diphosphate –glucuronosyltransferases
UHPLC	ultra high performance liquid chromtography
UV	ultraviolet
vd	volume of distribution
WHO	World Health Organisation
ZEA	zearalenone
RS	reactive species
USA	United States of America
PUFAs	polyunsaturated fatty acids
